# Personality and Humor Profiles in Rheumatoid Arthritis: A Latent Profile Analysis of Quality of Life and Medication Adherence

**DOI:** 10.7759/cureus.106692

**Published:** 2026-04-08

**Authors:** Parthena Alexandridou, Mary Gouva, Nektaria Zagorianakou, Zoe Konstanti, Anastasios Tzenalis, Eleni N Albani

**Affiliations:** 1 Laboratory of Child Care and Family Resilience, Department of Nursing, University of Patras, Patras, GRC; 2 Scientific Laboratory of Psychology and Person-Centered Care, School of Health Sciences, University of Ioannina, Ioannina, GRC; 3 Department of Nursing, University of Patras, Patras, GRC

**Keywords:** biopsychosocial model, health-related quality of life, humor styles, latent profile analysis, medication adherence, mental health, patient-centered care, personality traits, psychosocial profiling, rheumatoid arthritis

## Abstract

Background: Personality and humor styles are independently associated with mental health and health outcomes. However, the combined effects of these factors on health-related quality of life (HRQoL) and medication adherence remain under-explored from a person-centered perspective. Understanding psychological profiles may help clinicians identify patients at risk for poor adjustment and suboptimal adherence.

Aim: The aim of this study was to identify latent profiles characterized by Extraversion, Neuroticism, self-enhancing humor, and self-defeating humor, specifically among patients diagnosed with rheumatoid arthritis (RA). The study further investigated whether an adaptive profile distinguished itself from others in terms of HRQoL and medication adherence to inform rheumatology practice.

Methods: A cross-sectional study was conducted with a clinical sample of 200 adults diagnosed with RA, recruited via convenience sampling from the outpatient rheumatology clinic of the University General Hospital of Patras "Panagia i Voithia", and the General Hospital of Patras "Agios Andreas". Latent Profile Analysis (LPA) was utilized to identify distinct psychological subgroups. Differences in distal outcomes were examined using linear regression models adjusting for age.

Results: Four distinct and theoretically meaningful profiles emerged, accounting for significant variance in outcomes (R2=33.9% for mental HRQoL and 16.1% for adherence, representing moderate to large effect sizes). A profile characterized by high Extraversion and self-enhancing humor, combined with low Neuroticism and self-defeating humor, was identified as the most adaptive configuration. Notably, Neuroticism showed a strong and statistically significant negative association with mental HRQoL (r=−0.524, p<0.01), serving as a key distinguishing factor among the profiles. Planned comparisons indicated that the adaptive profile exhibited substantially higher mental HRQoL compared to other profiles, with more modest but significant advantages for physical HRQoL and medication adherence.

Conclusions: The study demonstrates that profiles defined by personality and humor are strongly associated with mental HRQoL in RA patients. These findings emphasize the importance of holistic psychological configurations and support the integration of psychological profiling as a clinically meaningful component of personalized, patient-centered rheumatology care.

## Introduction

Rheumatoid arthritis (RA) is a chronic, systemic autoimmune disease characterized by persistent inflammation, progressive joint destruction, and substantial functional impairment, leading to long-term disability and reduced quality of life [1,2]. Despite significant advances in pharmacological treatment and disease-modifying therapies, considerable variability remains in patients’ psychological adjustment, perceived health status, and treatment outcomes. Individuals living with RA consistently demonstrate higher rates of psychological distress and lower levels of psychological well-being compared to the general population, highlighting the importance of psychosocial determinants alongside biomedical factors [3].

Personality is one of the most stable psychological determinants influencing adaptation to chronic illness. Research has repeatedly shown that patients with RA tend to report lower levels of Extraversion and elevated levels of Neuroticism compared to normative populations [4,5]. These personality dimensions have clinically relevant implications. Higher Neuroticism has been associated with chronic emotional distress, heightened symptom perception, and maladaptive illness appraisal [6], whereas higher Extraversion has been linked to improved pain outcomes, greater social engagement, and more adaptive coping responses [7]. Extraversion and Neuroticism were selected as they are robust predictors of emotional regulation, stress reactivity, and health behavior across chronic disease populations.

Beyond personality traits, coping-related psychological processes also play a central role in shaping how individuals interpret and manage illness-related stressors. Humor, in particular, has attracted growing attention as a psychological resource influencing emotional coping and illness appraisal in chronic disease contexts [8]. Within rheumatology settings, humor has even been incorporated into nursing practice as a supportive strategy for reducing pain and anxiety among patients with RA [9]. Importantly, however, humor is not a unitary construct. Adaptive humor styles, including affiliative and self-enhancing humor, are associated with emotional resilience and effective regulation of stress, whereas maladaptive forms-especially self-defeating humor-have been linked to psychological vulnerability, negative self-evaluation, and emotional distress [10-13].

Most existing research examining personality or humor in health contexts has relied on variable-centered approaches that investigate independent associations between individual traits and outcomes while assuming population homogeneity. Although these approaches have generated valuable findings, they provide limited insight into how psychological characteristics coexist and interact within individuals. Person-centered methodologies offer an important complementary perspective by focusing on configurations of characteristics rather than isolated variables. Latent Profile Analysis (LPA), in particular, enables the identification of unobserved subgroups sharing similar psychological patterns, allowing adaptive and maladaptive outcomes to be understood as emerging from combinations of traits and coping styles rather than single dimensions in isolation. Such an approach may provide a more ecologically valid understanding of psychological resilience and vulnerability in chronic illness populations.

Previous person-centered investigations have demonstrated that distinct personality or humor configurations are associated with differential psychological and behavioral outcomes [14-16]. However, most studies have examined personality traits or humor styles separately and have predominantly relied on non-clinical samples. Within RA populations, person-centered analyses have primarily focused on disease activity or health-related quality of life profiles rather than broader psychological functioning [17,18]. Consequently, evidence integrating personality and humor within a latent profile framework remains limited, particularly regarding their simultaneous association with quality of life and medication adherence.

Despite advances in pharmacological management, variability in patient outcomes suggests that psychological factors remain a critical yet underutilized component of RA care. Identifying clinically meaningful psychological configurations may therefore represent a critical step toward fully integrated RA care.

The present study addresses this gap by adopting a person-centered analytic framework to examine how configurations of Extraversion, Neuroticism, self-enhancing humor, and self-defeating humor relate to mental health-related quality of life, physical health-related quality of life, and medication adherence in patients with RA. While previous research has utilized LPA to explore personality traits or coping mechanisms independently, the unique contribution of the present study lies in the integrated examination of stable personality traits alongside dynamic humor styles as specific coping strategies in a clinical RA population. By adopting this holistic person-centered approach, we move beyond isolated variable-centered analyses to identify how synergistic psychological configurations-rather than single traits-jointly influence both subjective health-related quality of life and behavioral medication adherence. This integration provides a more nuanced understanding of patient heterogeneity in rheumatology care. Specifically, this study aims to evaluate whether LPA can classify RA patients into distinct personality-humor profiles and to determine whether an adaptive psychological configuration is associated with more favorable mental health, physical health, and treatment adherence outcomes compared with alternative profiles.

## Materials and methods

Participants

The data collection was carried out during spring and summer 2025. The clinical sample consisted of 200 patients specifically diagnosed with rheumatoid arthritis (RA) (137 females, 63 males; mean age 55.6 years). Participants were recruited via convenience sampling from the outpatient rheumatology clinics of the University General Hospital of Patras "Panagia i Voithia" and the General Hospital of Patras "Agios Andreas". Participants had been living with RA symptoms for an average of 14.7 years (SD = 10.7). Eligibility criteria included a confirmed diagnosis of RA by a specialist, current use of prescribed medication, and the ability to comprehend self-report questionnaires. Participants were informed about the aims of the study and provided written informed consent prior to voluntary participation. Ethical approval was obtained from the Institutional Review Board of the University of Patras, Department of Nursing (Ref: 83/11-03-2025), and the study adhered to the Declaration of Helsinki.

Measures

Health-Related Quality of Life

Health-related quality of life was measured using the 36-Item Short Form Health Survey (SF-36) [18]. Internal consistency estimates for the SF-36 subscales in the present sample ranged from α=0.61 to 0.92, which is consistent with previous research and the validated Greek version of the instrument [19]. The validated Greek version was used with formal authorization. In the present study, the Mental Component Summary (MCS) and the Physical Component Summary (PCS) were utilized.

Medication Adherence

Medication adherence was assessed with the 8-item Morisky Medication Adherence Scale (MMAS) [20]. The scale evaluates both intentional and unintentional non-adherence behaviors. In the present study, the Greek validated version of the total MMAS score was used [21], with higher scores indicating higher levels of medication adherence. Documented proof of permission and licensing for the use of the MMAS-8 has been provided to the Editor.

Personality Traits

Personality traits were assessed using the Eysenck Personality Questionnaire (EPQ) [22] with the Greek validated version [23], used with appropriate licensing. The Extraversion (EXT) and Neuroticism (NEU) subscales were included in the analyses, as these dimensions are consistently associated with psychological well-being and health-related behaviors. Higher scores reflected higher levels of the corresponding personality trait.

Humor Styles

Humor styles were measured using the Humor Styles Questionnaire (HSQ) and especially the Greek validated version [12]. The subscales, Self-enhancing humor and Self-defeating humor, were included in the model. Self-enhancing humor represents an adaptive humor style, whereas self-defeating humor reflects a maladaptive style associated with psychological vulnerability. Higher scores indicated greater use of each humor style.

Procedure

Participants completed the questionnaires in paper format. All measures were administered in a single assessment session. Participants were instructed to answer honestly and were assured of the confidentiality and anonymity of their responses. The study was conducted in accordance with ethical standards for research involving human participants. Ethical approval was obtained from the Institutional Review Board of the University of Patras, Department of Nursing (Ref: 83/11-03-2025), and the study adhered to the Declaration of Helsinki.

Statistical analysis

Latent Profile Analysis (LPA) was conducted on the total sample of participants (N = 200), all of whom had complete data on Extraversion, Neuroticism, self-enhancing, and self-defeating humor styles. Latent profile solutions with three to five classes were estimated assuming equal variances across profiles and zero within-profile covariances. The optimal number of profiles was determined based on the Bayesian information criterion (BIC), the Akaike information criterion (AIC), and the entropy [24,25]. For each solution (three to six profiles), we calculated the coefficient of variation (CV) of BIC values across bootstrap samples, with CV < 5% indicating excellent stability [26]. Bootstrap analysis was conducted with 100 iterations, each using random resampling with replacement from the original dataset.

The relevance of the LPA solution was assessed using analysis of covariance, which evaluated between-group differences on medication adherence and HRQoL outcomes. Age, gender, and RA duration (years since RA diagnosis) were included as covariates in all models. These models were tested on 182 respondents, after omitting 1 missing value for age and 17 missing values for RA duration. Planned comparisons were evaluated using estimated marginal means derived from the fitted regression models in order to compare the most adaptive profile with the remaining profiles. Missing data were handled using listwise deletion, which was appropriate given that the missingness was minimal. Age, gender, and disease duration were included as covariates in the analysis to control for their well-documented influence on HRQoL and treatment adherence in rheumatoid arthritis populations. To ensure the stability of the latent profiles and avoid local maxima, the analysis followed standard iterative procedures with multiple random starting values, confirming the replicability of the four-class solution. The analysis was conducted using R software (R Foundation for Statistical Computing, Vienna, Austria) with the tidyLPA and emmeans packages [27-30]. This approach was employed to enhance the methodological robustness and replicability of the latent profile solution.

## Results

Descriptive statistics, internal consistency indices, and Pearson correlations among the study variables are presented in Table 1. The constructs demonstrated good internal consistency, with Cronbach’s α ranging from 0.63 to 0.81 for the primary predictors.

**Table 1:** Descriptive statistics, internal consistency coefficients, and Pearson correlations among study variables EPQ = Eysenck Personality Questionnaire; HSQ = Humor Styles Questionnaire; MCS = Mental Component Summary; PCS = Physical Component Summary; EXT = Extraversion; NEU = Neuroticism; ENH = Enhancing (Affiliative) Humor; SDF = Self-Defeating Humor; MMAS = Morisky Medication Adherence Scale; SF-36 = 36-Item Short Form Health Survey *: p < 0.05, **: p < 0.01

Variables	M (SD)	Cronbach’s α	Age	EPQ		HSQ		MCS	PCS
				EXT	NEU	ENH	SDF		
Age	55.6 (14.0)	-	-	-	-	-	-	-	-
EPQ									
EXT	12.5 (4.2)	0.810	-0.059	-	-	-	-	-	-
NEU	12.7 (4.3)	0.804	0.028	-0.266^**^	-	-	-	-	-
HSQ									
ENH	36.2 (8.0)	0.634	-0.008	0.325^**^	-0.069	-	-	-	-
SDF	25.8 (9.0)	0.683	-0.111	-0.023	0.241^**^	0.167^*^	-	-	-
SF-36									
MCS	41.8 (10.9)	-	-0.068	0.321^**^	-0.524^**^	0.081	-0.282^**^	-	-
PCS	39.3 (10.3)	-	-0.278^**^	0.131	-0.255^**^	0.102	-0.045	0.193^**^	-
MMAS	5.99 (2.0)	0.748	0.063	0.128	-0.212^**^	0.089	-0.124	0.389^**^	0.126

The constructs that participated in the model showed good internal consistency. Notably, a strong and statistically significant negative association was observed between Neuroticism and mental HRQoL (MCS) (r=−0.524, p<0.01), identifying it as the most potent correlate of psychological well-being in this RA cohort. Self-defeating humor was also negatively related to MCS (r=−0.282, p<0.01), whereas Extraversion showed positive associations (r=0.321, p<0.01). Medication adherence (MMAS) was moderately and positively correlated with MCS (r=0.389, p<0.01).

The mean medication adherence score was 5.99. According to the suggested classification, 72 of the 200 participants (36.0%) were classified as having low medication adherence (score < 6), 73 (36.5%) as having moderate adherence (score ≥ 6 and < 8), and 55 (27.5%) as having high adherence (score = 8). No statistically significant gender differences were observed for the variables included in the LPA analysis, with the exception of Neuroticism, where female participants reported significantly higher Neuroticism scores than male participants (13.4 ± 4.04 vs. 11.1 ± 4.59; t(197) = 3.460, p < 0.001).

Latent profile analysis

Although the three-class solution yielded a lower BIC, the four-class solution was retained due to better theoretical interpretability and classification quality (Table 2). In contrast, the five and six-class solutions showed higher BIC values and resulted in the emergence of small and potentially unstable classes. Accordingly, a four-profile solution was retained, providing a balance between classification quality and theoretical interpretability. The final models demonstrated substantial explanatory power, accounting for 33.9% of the variance in mental HRQoL and 16.1% in medication adherence, representing moderate to large effect sizes for the identified profiles. Alternative model specifications and robustness analyses, including sensitivity analyses and bootstrap resampling procedures, were conducted and are reported in Appendix A.

**Table 2:** Model fit indices and classification diagnostics for latent profile solutions BIC = Bayesian information criterion; AIC = Akaike information criterion

Classes	AIC	BIC	Entropy	p_min_	p_max_	Smallest class (%)
3	2227	2286	0.602	0.787	0.847	22.5
4	2226	2302	0.613	0.689	0.849	13.5
5	2234	2327	0.644	0.642	0.85	6.0
6	2204	2313	0.784	0.769	0.886	5.0

Table 3 presents the mean levels and standard deviations of Extraversion, Neuroticism, and humor styles characterizing the four latent profiles. 

**Table 3:** Descriptive Statistics of the variables across latent profiles EXT = Extraversion; NEU = Neuroticism; ENH = Enhancing (Affiliative) Humor; SDF = Self-Defeating Humor

Class	Label	N	EXT	NEU	ENH	SDF
1	Self-critical	63	13.1 (3.51)	16.6 (1.93)	39.6 (6.83)	32.1 (8.45)
2	Vulnerable	27	6.26 (2.40)	16.5 (2.41)	26.6 (6.55)	21.1 (6.84)
3	Moderate	69	11.9 (3.11)	10.6 (2.30)	34.5 (6.19)	24.8 (7.40)
4	Adaptive	41	16.4 (2.04)	7.68 (2.81)	40.1 (7.04)	21.0 (8.18)

**Figure 1:**
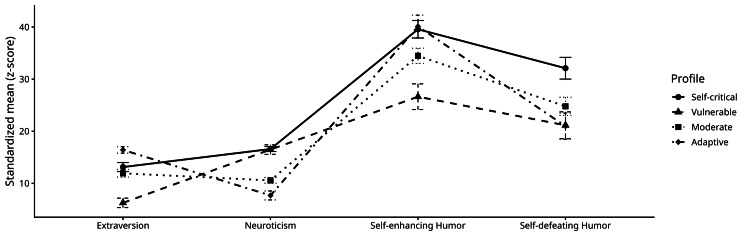
Standardized mean levels (z-scores) of Extraversion, Neuroticism, self-enhancing humor, and self-defeating humor across the four latent personality–humor profiles Profile 1 = Self-critical; Profile 2 = Vulnerable; Profile 3 = Moderate; Profile 4 = Adaptive Error bars represent 95% confidence intervals

The profiles demonstrated differences across all indicators (Figure 1). Profile 4 (Adaptive) was characterized by high Extraversion and humor, combined with particularly low Neuroticism and self-defeating humor, suggesting a broadly adaptive and resilient configuration. In contrast, Profile 2 (Vulnerable) exhibited markedly lower Extraversion, increased Neuroticism, and lower use of humor. Profiles 1 and 3 represented intermediate configurations. Profile 1 (Self-critical) combined moderate Extraversion with relatively high Neuroticism and elevated self-defeating humor, whereas Profile 3 (Moderate) was distinguished by lower Neuroticism and moderate levels of Extraversion and humor styles, reflecting a more normative pattern.

The latent profiles did not differ in patients’ age (p = 0.640), RA duration (p = 0.169), or odds of hospitalization due to RA (p = 0.768) (Table 4). In contrast, a significant association of gender and class was observed, due to a greater than expected representation of men in the Adaptive group as well as a greater than expected representation of women in the Self-critical group.

**Table 4:** Comparison of demographic and study variables across latent profiles RA = rheumatoid arthritis; HRQoL = health-related quality of life; MCS = Mental Component Summary; PCS = Physical Component Summary; MMAS = Morisky Medication Adherence Scale Profile 1 = Self-critical; Profile 2 = Vulnerable; Profile 3 = Moderate; Profile 4 = Adaptive Values represent means and standard deviations unless otherwise indicated

Class	Self-critical	Vulnerable	Moderate	Adaptive	Test statistic
N	63	27	69	41	-
Demographics					
Age (M ± SD)	54.2 (13.5)	57.9 (13.7)	56.4 (15.0)	54.7 (13.3)	F(3,196) = 0.562, p = .640
Gender					
Men (63)	16	3	25	19	χ²(3) = 11.20, p = .011
Women (137)	47	24	44	22	
Medical					
RA years (M ± SD)	16.9 (12.1)	12.5 (12.1)	13.1 (8.58)	15.1 (9.95)	F(3,192) = 1.697, p = .169
RA hospitalization	28 (44.4%)	11 (40.7%)	29 (42.0%)	14 (34.1%)	χ²(3) = 1.140, p = .768
HRQoL and Adherence					
MCS	−1.39 (0.817)	−1.31 (1.12)	−0.815 (0.884)	0.381 (0.761)	F(3,196) = 37.47, p < .001
PCS	−1.16 (0.923)	−1.44 (1.18)	−1.07 (1.00)	−0.682 (1.04)	F(3,196) = 3.40, p = .019
MMAS	5.83 (1.96)	5.19 (2.10)	5.97 (1.95)	6.82 (1.68)	F(3,196) = 4.29, p = .006

Further differences in distal outcomes across latent profiles were examined using linear regression models adjusted for age, gender, and RA duration. The regressions were conducted on 182 participants, after excluding 17 cases with missing RA duration values and one case with a missing age value. Full regression results are presented in Appendix B.

To address the primary theoretical question, the most Adaptive profile (Profile 4) was compared with the remaining profiles using planned comparisons based on estimated marginal means. The corresponding results are summarized in Table 5.

**Table 5:** Comparison of distal outcomes between the Adaptive and the remaining profiles MCS = Mental Component Summary; PCS = Physical Component Summary; MMAS = Morisky Medication Adherence Scale; RA = rheumatoid arthritis Estimates represent adjusted mean differences between Profile 4 (Adaptive) and the average of Profiles 1–3 (Self-critical, Vulnerable, and Moderate), controlling for age, gender, and RA duration

Outcome	Estimate	95% CI	SE	t	p
MCS	4.49	3.47 – 5.51	0.52	8.67	< .001
PCS	1.16	0.065 – 2.25	0.55	2.10	.004
MMAS	3.45	1.19 – 5.70	1.14	3.02	.003

The adaptive latent profile differed markedly from the remaining profiles in mental health-related quality of life. Specifically, the adaptive profile exhibited substantially higher MCS scores compared to the average of Self-critical, Vulnerable, and Moderate profiles (Profiles 1-3; estimate = 4.49, SE = 0.52, t(175) = 8.67, p < 0.001), after adjusting for age, gender, and RA duration (Table 5). 

**Figure 2:**
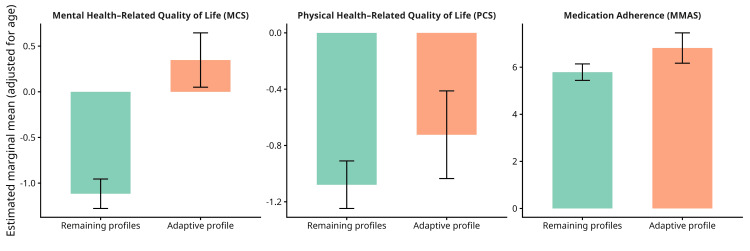
Estimated marginal means (±95% confidence intervals) for mental health–related quality of life (MCS), physical health–related quality of life (PCS), and medication adherence (MMAS), comparing the Adaptive profile with the remaining profiles MCS = Mental Component Summary; PCS = Physical Component Summary; MMAS = Morisky Medication Adherence Scale

Furthermore, a significant yet modest advantage of the adaptive profile in physical health-related quality of life was reported. Specifically, Profile 4 (Adaptive) showed higher PCS scores compared to the combined set of the remaining profiles (estimate = 1.16, SE = 0.55, t(175) = 2.10, p = 0.004). Finally, planned comparisons demonstrated that Profile 4 (Adaptive) exhibited significantly higher MMAS scores compared to the remaining profiles (estimate = 3.45, SE = 1.14, t(175) = 3.02, p = .003). Overall, the adaptive personality-humor profile consistently demonstrated more favorable mental health, physical health, and adherence outcomes compared to the remaining profiles (Figure 2).

These profiles appear to represent clinically interpretable psychological patterns rather than purely statistical groupings. This finding supports their potential clinical applicability and extends beyond statistical classification toward clinically relevant patient stratification.

## Discussion

The present study applied a person-centered analytic approach to identify latent personality-humor profiles among patients with rheumatoid arthritis and to examine their associations with mental and physical health-related quality of life as well as medication adherence. Four empirically and theoretically interpretable profiles emerged, characterized by distinct configurations of Extraversion, Neuroticism, self-enhancing humor, and self-defeating humor. Importantly, these profiles were not differentiated by demographic or disease-related characteristics such as age or disease duration, yet they demonstrated significant differences across key health outcomes, with particularly pronounced effects observed for mental health-related quality of life.

A central finding of the study was the consistent advantage of the adaptive profile, defined by high Extraversion and self-enhancing humor combined with low Neuroticism and reduced reliance on self-defeating humor. Individuals belonging to this profile exhibited markedly superior mental HRQoL and significantly higher medication adherence compared to the remaining profiles. The strongest effect was observed for mental health outcomes, supporting extensive literature demonstrating that low Neuroticism and higher Extraversion are robust predictors of psychological well-being, emotional stability, and resilience [10,31,32]. The present findings extend this body of work by demonstrating that psychological outcomes are not adequately explained by isolated traits alone but rather emerge from integrated personality-coping configurations.

Personality has long been recognized as a central determinant of health-related functioning across medical conditions [33-36]. Within the context of rheumatoid arthritis, earlier investigations suggested the existence of personality differences compared with healthy populations or individuals with other chronic diseases, sometimes interpreted as psychological consequences of long-term illness burden [5,37]. However, subsequent evidence challenged the notion of a distinct “rheumatoid personality,” demonstrating comparable personality structures across chronic illness groups [38]. The present findings contribute to this ongoing debate by suggesting that adaptive psychological functioning in rheumatoid arthritis may reflect enduring dispositional resources rather than disease-induced personality change, consistent with lifespan models of personality stability. The absence of associations between profile membership and disease duration or hospitalization history further supports the interpretation that psychological resilience patterns likely precede illness onset and subsequently shape adjustment trajectories.

Differences in physical health-related quality of life were comparatively modest. Although individuals within the adaptive profile demonstrated significantly higher PCS scores, the magnitude of this effect was smaller than that observed for mental HRQoL. This pattern aligns with theoretical models proposing that personality and coping processes exert stronger and more direct effects on psychological outcomes, while physical health may be influenced more indirectly through behavioral regulation, stress physiology, and long-term lifestyle processes [39]. Thus, personality-humor configurations may not directly modify disease pathology but may influence how patients experience, interpret, and function within the constraints imposed by chronic illness.

Medication adherence represents a complex behavioral outcome shaped by medical, contextual, interpersonal, and psychological determinants [40,41]. Previous research has consistently demonstrated positive associations between Extraversion and adherence behaviors and negative associations between Neuroticism and treatment engagement [42-44]. The present study expands these findings by demonstrating that adherence is better understood within broader psychological configurations rather than single personality dimensions. Patients characterized by the adaptive profile reported significantly higher adherence, suggesting that emotion regulation capacity, optimistic appraisal, and constructive humor may facilitate sustained engagement with treatment demands and healthcare interactions.

Collectively, these findings support a shift from variable-centered psychological assessment toward integrated psychological profiling in rheumatoid arthritis care. Rather than evaluating isolated traits as background psychological characteristics, clinicians may benefit from recognizing coherent psychological patterns that reflect patients’ adaptive or vulnerable coping orientations. Such profiling may assist in identifying individuals at increased risk for emotional distress, reduced engagement with care, or poorer adjustment early in the disease trajectory, thereby enabling timely psychosocial intervention.

Importantly, the results suggest that personality-humor configurations may function as clinically meaningful stratification markers within chronic disease management. This perspective aligns with contemporary person-centered medicine approaches, emphasizing individualized care pathways that integrate biological, psychological, and behavioral dimensions of health. Psychological profiling offers a framework for enhancing communication strategies, strengthening therapeutic alliance, and tailoring supportive interventions according to patient needs.

The present study demonstrates that latent profiles defined by personality traits and humor styles are strongly associated with mental health-related quality of life and, to a lesser extent, with physical health-related quality of life and medication adherence in patients with rheumatoid arthritis. By applying a person-centered analytic framework, the study highlights the added value of examining holistic psychological configurations rather than isolated variables. The findings reinforce the importance of integrating psychological understanding into chronic disease management and position personality-humor profiles as potential indicators of resilience and vulnerability within clinical practice. Taken together, the findings position psychological profiling not as an adjunct perspective but as an integral dimension of comprehensive rheumatoid arthritis care.

Clinical implications

The present findings offer clinically meaningful implications for the assessment and management of individuals living with chronic health conditions, particularly rheumatoid arthritis. The identification of distinct personality-humor profiles highlights that patients differ not merely in isolated psychological traits but in integrated psychological configurations that influence emotional adjustment, perceived quality of life, and treatment-related behaviors.

From a clinical perspective, the Adaptive profile - characterized by low Neuroticism, high Extraversion, and greater use of self-enhancing humor - appears to function as a psychological resilience pattern. Patients presenting this configuration may possess emotional and interpersonal resources that facilitate coping with chronic illness demands, promote constructive engagement with healthcare professionals, and support sustained participation in treatment plans.

These findings suggest that routine clinical assessment may benefit from incorporating brief psychological screening procedures capable of identifying broader personality-coping patterns rather than single risk factors alone. Given the cross-sectional design, these findings should be viewed as descriptive patterns of patient heterogeneity rather than evidence of a causal relationship between personality and clinical outcomes. Recognizing patients who belong to more vulnerable psychological profiles may allow healthcare providers to implement early supportive interventions aimed at reducing emotional burden and preventing disengagement from treatment.

The observed association between adaptive profiles and higher medication adherence further indicates that adherence behavior may partly reflect motivational and relational processes. Patients with adaptive psychological configurations may experience greater treatment self-efficacy, lower illness-related distress, and stronger therapeutic alliance with clinicians. Conversely, individuals characterized by higher Neuroticism or maladaptive humor patterns may require targeted psychosocial support, including enhanced communication strategies, trust-building approaches, psychoeducation, and stress-management interventions.

Overall, the findings support a shift toward psychologically informed, person-centered care models in chronic disease management. Integrating personality and coping profiles into clinical decision-making may help clinicians tailor communication style, optimize adherence support, and promote holistic patient well-being beyond purely biomedical indicators. Importantly, psychological profiling does not aim to label patients but to support individualized care pathways. Integrating psychological understanding into medical practice may enhance empathy, communication effectiveness, and long-term therapeutic engagement.

Limitations

The cross-sectional design precludes causal inferences regarding the directionality of associations between personality-humor profiles and health-related outcomes. Thus, longitudinal designs are needed to determine whether adaptive profiles are associated with improvements in mental health, physical health, or adherence, or whether these outcomes in turn shape personality-related processes. In addition, replication in more diverse and clinically heterogeneous samples would help establish the generalizability of the identified profiles. The relatively small size of certain identified subgroups, such as the Vulnerable profile (n=27), may limit the statistical power to detect smaller effects and could potentially impact the stability of the profile classification. Furthermore, the use of self-report measures may introduce response biases, such as social desirability, which should be considered when interpreting the findings. The study’s reliance on convenience sampling from specific clinical settings may introduce selection bias, potentially limiting the representativeness of the findings for the wider RA community. Moreover, the expression of humor styles and personality traits may be subject to cultural specificity; thus, the patterns identified in this Greek RA population should be validated in different sociocultural settings to ensure external validity. Additionally, the predominance of female participants, while reflecting the typical epidemiology of RA, may have influenced the distribution of personality and humor styles; therefore, gender should be considered as a potential confounder in future larger-scale studies.

## Conclusions

The present study demonstrates that distinct latent profiles defined by personality traits and humor styles are meaningfully associated with health-related quality of life and medication adherence. By applying a person-centered analytic framework and planned comparisons, the findings highlight the central role of adaptive psychological configurations, characterized by low Neuroticism, high Extraversion, and adaptive humor, in promoting more favorable health-related outcomes. Rather than viewing psychological characteristics as peripheral to medical care, the present findings support the integration of psychological profiling as a core component of holistic chronic disease management.

## Appendices

Appendix A

Sensitivity and Robustness Analyses

A. Additional Latent Profile Analysis (LPA) specifications incorporating all Eysenck Personality Questionnaire (EPQ) and Humor Styles Questionnaire (HSQ) subscales were examined, suggesting that increased indicator complexity primarily reflected a general adaptive-maladaptive dimension rather than distinct personality-humor configurations. Accordingly, the more interpretable and theoretically focused model was retained.

**Table 6:** Sensitivity analysis of LPA solutions under alternative model specifications LPA = Latent Profile Analysis; EXT = Extraversion; NEU = Neuroticism; ENH = Enhancing (Affiliative) Humor; SDF = Self-Defeating Humor; AFF = Affiliative Humor; AGR = Aggressive Humor; HSQ = Humor Styles Questionnaire; EPQ = Eysenck Personality Questionnaire; vars = variables

Model	Indicators	Solution
EXT, NEU, ENH, SDF	4	4 classes
+ AFF	5	3 classes
+ AFF + AGR	6	3 classes
All 4 HSQ subscales	4	3 classes
All EPQ, HSQ subscales (8 vars)	8	2 classes

B. Bootstrap analysis was conducted using resampling with replacement. Results indicated that Bayesian information criterion (BIC) and entropy values were highly stable across bootstrap samples, and the relative ordering of candidate solutions was fully consistent with the full-sample analysis.

**Table 7:** Bootstrap stability of information criteria and classification indices across latent profile solutions BIC = Bayesian information criterion; AIC = Akaike information criterion; CV = coefficient of variation

Profiles	3	4	5	6
Selected solution				
BIC	2286	2302	2327	2313
AIC	2227	2226	2234	2204
Entropy	0.602	0.613	0.644	0.784
Min profile percent (N)	22.5% (45)	13.5% (27)	6% (12)	5% (10)
Bootstrap analysis				
BIC (M (SD))	2250 (41.1)	2256 (41.1)	2262 (41)	2269 (38,5)
BIC CV	1.8%	1.8%	1.8%	1.7%
AIC (M(SD))	2190 (41.1)	2180 (41.1)	2169 (41)	2161 (38.5)
AIC CV	1.9%	1.9%	1.9%	1.8%
Entropy (M(SD))	0.698 (0.06)	0.726 (0.07)	0.757 (0.06)	0.775 (0.05)
Entropy min	0.541	0.524	0.573	0.651
Entropy max	0.812	0.857	0.884	0.874

Appendix B

Linear Regression Results

The following tables present the full linear regression results for all outcome variables.

**Table 8:** Linear regression results for MCS MCS = Mental Component Summary; RA = rheumatoid arthritis F(6, 175) = 15.126, R2 = 0.341

					95% CI	
	B	SE	t	p	Lower	Upper
(Intercept)	-1.224	0.298	-4.105	<0.001	-1.812	-0.635
Class2	0.129	0.218	0.59	0.556	-0.302	0.559
Class3	0.492	0.166	2.97	0.003	0.165	0.819
Class4	1.703	0.191	8.914	<0.001	1.326	2.08
Age	-0.002	0.005	-0.385	0.701	-0.012	0.008
Gender (Woman)	-0.021	0.147	-0.14	0.889	-0.311	0.27
RA duration	-0.001	0.007	-0.166	0.868	-0.014	0.012

**Table 9:** Linear regression results for PCS PCS = Physical Component Summary; RA = rheumatoid arthritis F test: F(6, 175) = 4.612, p < 0.001, R2 = 0.137

					95% CI	
	B	SE	t	p	Lower	Upper
(Intercept)	-0.012	0.32	-0.036	0.971	-0.642	0.619
Class2	-0.162	0.234	-0.692	0.49	-0.623	0.3
Class3	0.006	0.178	0.034	0.973	-0.345	0.357
Class4	0.335	0.205	1.636	0.104	-0.069	0.739
Age	-0.014	0.005	-2.589	0.01	-0.025	-0.003
Gender (Woman)	-0.455	0.158	-2.879	0.004	-0.766	-0.143
RA duration	-0.002	0.007	-0.252	0.801	-0.016	0.012

**Table 10:** Linear regression results for MMAS MMAS = Morisky Medication Adherence Scale; RA = rheumatoid arthritis F test: F(6, 175) = 2.431, p = 0.009, R2 = 0.077

					95% CI	
	B	SE	t	p	Lower	Upper
(Intercept)	5.171	0.658	7.854	<0.001	3.872	6.471
Class2	-0.694	0.481	-1.443	0.151	-1.644	0.256
Class3	-0.079	0.366	-0.217	0.829	-0.802	0.643
Class4	0.891	0.422	2.112	0.036	0.058	1.723
Age	0.019	0.011	1.705	0.09	-0.003	0.042
Gender (Woman)	-0.343	0.325	-1.054	0.293	-0.984	0.299
RA duration	-0.01	0.014	-0.707	0.481	-0.039	0.018
